# Chloroquine modulates inflammatory autoimmune responses through Nurr1 in autoimmune diseases

**DOI:** 10.1038/s41598-019-52085-w

**Published:** 2019-10-29

**Authors:** Tae-Yoon Park, Yongwoo Jang, Woori Kim, Joon Shin, Hui Ting Toh, Chun-Hyung Kim, Ho Sup Yoon, Pierre Leblanc, Kwang-Soo Kim

**Affiliations:** 1 Molecular Neurobiology Laboratory, Department of Psychiatry and McLean Hospital, Harvard Medical School, 115 Mill Street, Belmont, Massachusetts 02478 USA; 2 School of Biological Sciences, Nanyang Technological University, 50 Nanyang Avenue, Singapore, 639798 Singapore; 3 Program in Neuroscience and Harvard Stem Cell Institute, McLean Hospital, Harvard Medical School, Belmont, MA 02478 USA

**Keywords:** Autoimmunity, NMR spectroscopy

## Abstract

For over a half-century the anti-malarial drug chloroquine (CQ) has been used as a therapeutic agent, alone or in combination, to treat autoimmune diseases. However, neither the underlying mechanism(s) of action nor their molecular target(s) are well defined. The orphan nuclear receptor Nurr1 (also known as NR4A2) is an essential transcription factor affecting the development and maintenance of midbrain dopaminergic neurons. In this study, using *in vitro* T cell differentiation models, we demonstrate that CQ activates T_REG_ cell differentiation and induces Foxp3 gene expression in a Nurr1-dependent manner. Remarkably, CQ appears to induce Nurr1 function by two distinct mechanisms: firstly, by direct binding to Nurr1’s ligand-binding domain and promoting its transcriptional activity and secondly by upregulation of Nurr1 expression through the CREB signaling pathway. In contrast, CQ suppressed gene expression and differentiation of pathogenic T_H_17 cells. Importantly, using a valid animal model of inflammatory bowel disease (IBD), we demonstrated that CQ promotes Foxp3 expression and differentiation of T_REG_ cells in a Nurr1-dependent manner, leading to significant improvement of IBD-related symptoms. Taken together, these data suggest that CQ ameliorates autoimmune diseases via regulating Nurr1 function/expression and that Nurr1 is a promising target for developing effective therapeutics of human inflammatory autoimmune diseases.

## Introduction

Originally developed as anti-malarial drugs, chloroquine (CQ) as well as hydroxychloroquine (HCQ) have become part of the standard components of treatment for patients suffering from autoimmune diseases such as rheumatoid arthritis (RA) and systemic lupus erythematosus (SLE)^1–3^. Numerous investigations into CQ/HCQ’s potential roles led to the proposition of diverse candidate mechanisms of action, including inhibition of immunological processes such as antigen presentation, proinflammatory cytokine expression, and inhibition of Toll-like receptors and NLRP3 inflammasome^4–7^. In particular, CQ was recently shown to increase the number of T_REG_ cells and rebalance T_H_17/T_REG_-mediated immunity, strongly suggesting that CQ/HCQ regulate autoimmune disease by modulating T cell subset function and/or differentiation^8,9^. Despite these progresses, neither precise mechanisms nor the identity of molecular targets underlying CQ/HCQ’s roles in autoimmune diseases are well understood.

Nurr1 is an orphan nuclear receptor that plays a key role in the development and maintenance of midbrain dopamine neurons^10,11^. Moreover, Nurr1 was reported to protect midbrain dopamine neurons by its anti-inflammatory effects via recruiting the CoREST corepressor complex and removing NF-κB from the pro-inflammatory gene promoters in microglia and astrocytes^12^. Interestingly, Nurr1 was also found to bind to the regulatory regions of the Foxp3 gene and directly regulates its expression^13^. Furthermore, ectopic expression of Nurr1 in naïve CD4^+^ T cells resulted in induction of the T_REG_ cell developmental program and inactivation of Nurr1 and other NR4A members led to severe interference with T cell development, strongly supporting Nurr1’s critical roles in T cell homeostasis^14^.

Recently, we reported that the transcriptional activity of Nurr1 can be regulated by CQ and amodiaquine through Nurr1’s ligand-binding domain (LBD)^15^, prompting us to hypothesize that CQ could modulate autoimmune diseases via regulation of Nurr1’s function in T cells. To address this hypothesis, we used *in vitro* T cell differentiation models and investigated the potential mechanisms of CQ’s functional effects. We found that CQ not only directly binds to Nurr1-LBD to increase its transcriptional activity, but also it can increase Nurr1 expression in T cells through CREB, thereby enhancing Foxp3 expression and inducing T_REG_ cells differentiation. In contrast, CQ showed inhibitory effects on gene expression and differentiation of pathogenic T_H_17 cells, suggesting that CQ exhibits T cell subset-specific functional effects. In addition, we investigated a mouse model of inflammatory bowel disease (IBD) which is a chronic autoimmune disease of the colon and small intestine characterized by immune-mediated inflammation, diarrhea, rectal bleeding, swollen and damaged tissues of the digestive tract^16,17^. The dextran sulfate sodium (DSS)-induced mouse is a well-established model for studying IBD pathogenesis and developing novel therapies^18^. In particular, we chose this animal model to study the functional link between CQ and Nurr1 in autoimmune diseases because T cells’ essential roles are well validated for the development and continuation of the IBD disease process^19,20^. Using this DSS-induced mouse model, we showed that CQ can effectively improve symptoms of IBD in a Nurr1-dependent manner. Based on these data, we propose that targeting the CQ-Nurr1 interaction is a fundamental and effective strategy for the development of therapeutic agents for autoimmune diseases.

## Results

### CQ regulates T_REG_ cell differentiation through a Nurr1-dependent mechanism

**Figure 1:**
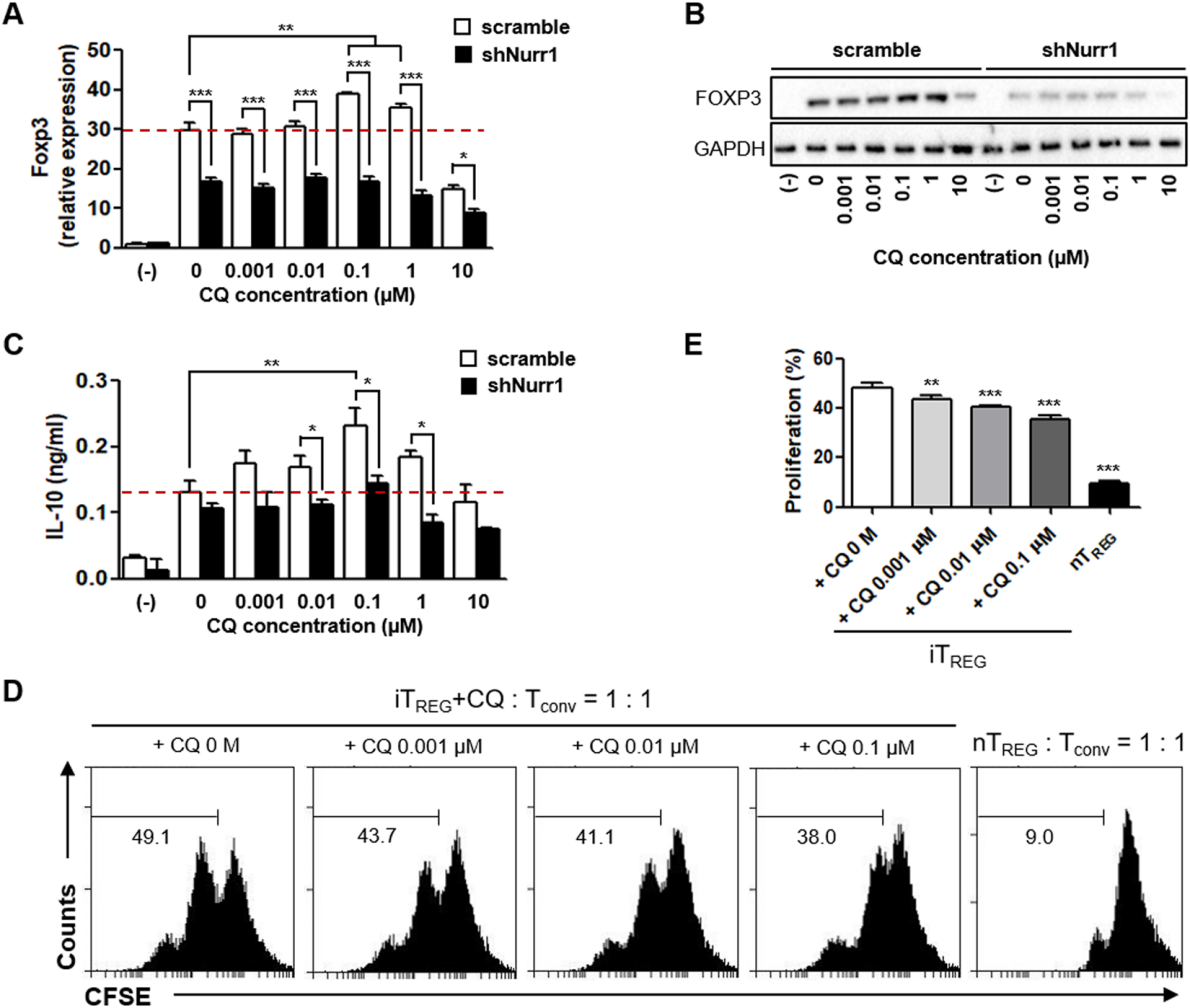
Nurr1-dependent regulation of iT_REG_ differentiation by CQ. Mouse primary naïve CD4^+^CD25^−^CD62L^high^ T cells were transfected with lenti-scramble- or lenti-shNurr1-plasmid. Cells were treated with CQ (0.001~10 μM) and stimulated with plate-bound anti-CD3 and soluble anti-CD28 antibodies for 96 h under iT_REG_-polarizing conditions. (**A**,**B**) The level of Foxp3 mRNA (**A**) or protein (**B**) expression was determined by quantitative real-time PCR or western blot, normalized with GAPDH. (**C**) The level of IL-10 in the culture media was analyzed by ELISA. (**D**) *In vitro* T_REG_ suppression assays based on CFSE dilution by T_conv_ cells proliferating in the presence of CQ treated iT_REG_ cells for 48 h and analyzed with flow cytometry. (**E**) Quantitation of the percentage ± SEM of proliferation of T_conv_ in the presence of iT_REG_ with or without CQ treatment. These experiments were repeated three times in triplicate using independently prepared samples. Each error bar represents means ± s.e.m. *P < 0.05, **P < 0.01, ***P < 0.001.

To address whether CQ regulates T_REG_ differentiation in a Nurr1-dependent manner, naïve CD4^+^CD25^−^CD62L^high^ T cells were isolated from C57BL/6 mice and transfected with a lentiviral shNurr1 or scramble vector, and then activated with anti-CD3 and CD28 antibodies under induced T_REG_ (iT_REG_)-polarizing condition in the presence of increasing doses of CQ. CQ treatment (up to 1 µM) increased Foxp3 and IL-10 expression (Fig. 1A–C) in a similar pattern to Nurr1 (Supplementary Fig. S1A). In addition, CQ treatment dose-dependently enhanced T_REG_’s suppressive activity as demonstrated in a T_REG_ suppression assay (Fig. 1D,E). When 10 µM CQ was used, expression of these genes (including Nurr1) was down-regulated, probably due to CQ’s cytotoxic effects. Indeed, treatment of mouse primary naïve T cells with CQ at concentrations of 10 and 100 µM resulted in significant reduction in the total cell number and specific gene expression (Supplementary Fig. S2A–C). In line with these results, it has been reported that high concentrations of CQ inhibit the activities of human CD4^+^ T cells^21^ as well as other cell types such as monocytes/macrophages^22,23^. Knocking down Nurr1 expression by transfecting differentiating T cells with lenti-shNurr1 plasmid (Supplementary Fig. S1A), inhibited up-regulation of Foxp3 gene and protein expression by CQ treatment (Fig. 1A,B). Thus, these data suggest that at concentrations ranging from 0.001 to 1 µM CQ regulates T_REG_ cell differentiation and increases expression of the Foxp3 gene in a Nurr1-dependent manner but shows cytotoxicity at concentration above 10 µM. In addition, Nurr1 knock-down inhibited CQ’s up-regulation of IL-10 expression (Fig. 1C). However, we observed modest up-regulation of IL-10 expression at 0.1 µM CQ, which may be due to incomplete knock-down of Nurr1. Alternatively, additional factor(s) may be involved in CQ’s up-regulation of IL-10 gene expression.

### CQ regulates pathogenic T_H_17 cell differentiation through a Nurr1-independent mechanism

**Figure 2:**
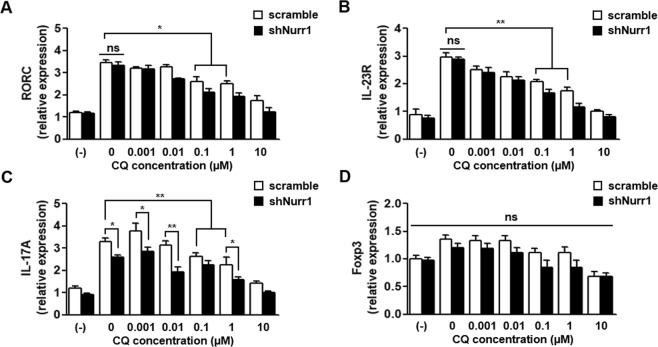
Nurr1-independent regulation of pT_H_17 differentiation by CQ. Mouse primary naïve CD4^+^CD25^−^CD62L^high^ T cells were transfected with lenti-scramble- or lenti-shNurr1-plasmid. Cells were treated with CQ (0.001~10 μM) and stimulated with plate-bound anti-CD3 and soluble anti-CD28 antibodies for 72 h under pT_H_17-polarizing conditions. (**A**–**C**) The levels of RORγt (**A**), IL-23R (**B**) or IL-17A (**C**) mRNA expression were determined by quantitative real-time PCR and normalized with GAPDH. (**D**) The levels of Foxp3 mRNA expression were determined by quantitative real-time PCR and normalized with GAPDH. These experiments were repeated three times in triplicate using independently prepared samples. Each error bar represents means ± s.e.m. *P < 0.05, **P < 0.01, ***P < 0.001.

Next, we examined the effect of CQ on pathogenic T_H_17 (pT_H_17) cell differentiation, which have opposite effects from T_REG_. CQ significantly decreased the expression of key marker genes of pT_H_17 (i.e., RORγt, IL-23R, and IL-17A) under pT_H_17-polarizing condition in a concentration-dependent manner (Fig. 2A–C). When Nurr1 expression was knocked down (Supplementary Fig. S1B), expression of IL-17A protein was reduced as was the number of CD4^+^ IL-17A^+^ T cells (Supplementary Fig. S3A–C), which is in agreement with the previous Nurr1 knockout study^24^. However, Nurr1 knockdown did not affect the expression of RORγt and IL-23R (Fig. 2A,B), suggesting that downregulation of RORγt and IL-23R genes is Nurr1-independent. We next tested how CQ treatment affects the expression of Foxp3 and Nurr1 during the pT_H_17 differentiation process and regulates the pathogenic properties of pT_H_17 cells. Foxp3 expression was unaffected by treatment with CQ and by Nurr1 knockdown (Fig. 2D). In agreement with these data, previous work showed that Foxp3 expression is very limited under pathogenic pT_H_17 differentiation conditions due to the lack of chromatin remodeling of Foxp3 via TGFβ signaling^25,26^. In addition, under T cell activation conditions, Foxp3 expression was not induced in spite of high Nurr1 expression resulting from CQ treatment (Supplementary Fig. S4A,B). Finally, we examined the effects of CQ treatment on gene expression during T_H_1 and T_H_2 cell differentiation where TGFβ signaling is also absent. As shown in Supplementary Fig. S4C,D, expression of Nurr1 and Foxp3 as well as the marker gene of T_H_1 and T_H_2 cell (Tbet and GATA3, respectively) was unaffected by CQ treatment. Taken together, the effect of CQ-Nurr1 interaction and function appears to be T cell subset-specific and was most evident during T_REG_ cell differentiation.

### CQ physically binds to Nurr1

**Figure 3:**
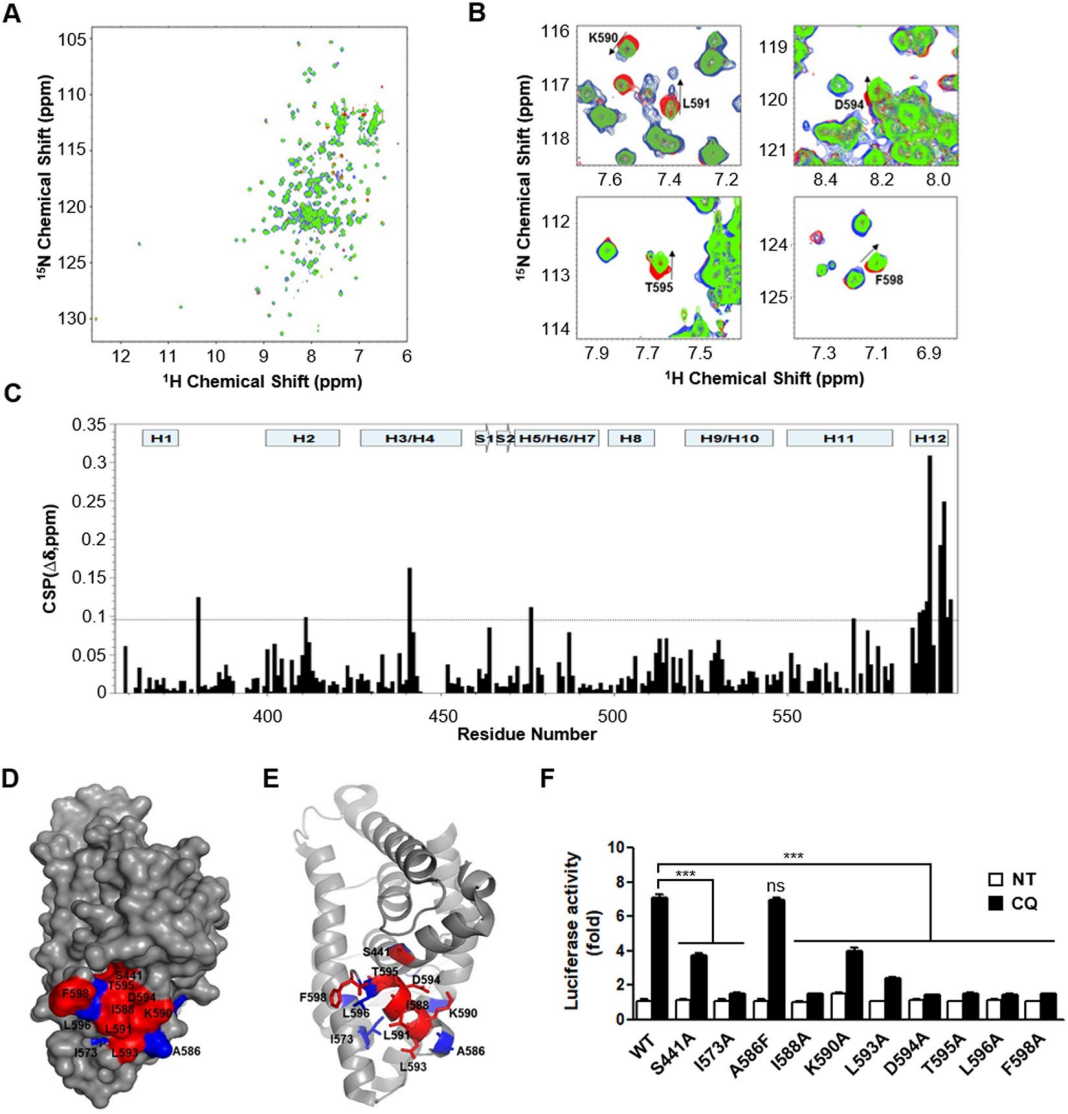
CQ interacts with Nurr1-LBD and directly regulates Nurr1 transcriptional activity. NMR titration experiments of Nurr1-LBD with CQ. (**A**) Overlay of ^1^H-^15^N HSQC spectra of uniformly ^15^N labeled Nurr1-LBD (red) and Nurr1-LBD in the presence of CQ at molar ratio of 1 to 2.5 (green) and 1 to 5 (blue). (**B**) Expanded views of chemical shift perturbations upon CQ binding. The perturbations of chemical shifts from free Nurr1-LBD (red) to CQ bound forms (green and blue) are indicated by arrows. (**C**) Chemical Shift Perturbation Plot of Nurr1-LBD upon CQ binding at molar ratio of 1 (Nurr1-LBD) to 5 (CQ). The difference in chemical shifts was calculated using the following formula, Δδ = [(^1^H_free_ − ^1^H_bound_)2 + (^15^N_free_ − ^15^N_bound_)^2^)]^1/2^. Interaction site mapping of Nurr1-LBD and CQ based on the NMR data. (**D**) Surface mapping of CQ binding site and interaction residues on Nurr1-LBD based on ^1^H-^15^N HSQC titration data. Perturbed amino acid residues are displayed according to their chemical shift perturbation: red (Δδ > 0.1), blue (0.8 < Δδ < 0.1). (**E**) Ribbon representation of mapping data in Fig. 3D. Perturbed amino acid residues were displayed by stick representation and chemical shift values were used in the same manner as in Fig. 3D. (**F**) Impact of Nurr1-LBD point mutation on CQ’s effect on Nurr1 transcriptional activity. Luciferase activities were diminished in full length mutant Nurr1 transfected HEK293 cell lines compared to wild-type Nurr1 transfected control. Each error bar represents means ± s.e.m. ***P < 0.001. NT, non-treated condition.

Our findings strongly suggest that CQ acts through Nurr1 in order to effect its anti-inflammatory function in autoimmune diseases. To further understand CQ’s regulation of Nurr1 function we investigated the interaction between CQ and Nurr1 using nuclear magnetic resonance spectroscopy (NMR). Upon addition of CQ, a number of amino acid residues on Nurr1-LBD showed significant chemical shift perturbations on two-dimensional ^1^H-^15^N Heteronuclear Single Quantum Correlation (HSQC) spectra of the ^15^N-labeled Nurr1-LBD. These perturbations were primarily located at the C-terminal helix α12 including S441, I573, A586, I588, K590, L593, D594, T595, L596, and F598 (Fig. 3A,B). Overlay of the free and ligand-bound spectra of Nurr1-LBD revealed that the perturbed residues are mainly located in the helix α12 region (I573, A586, I588, K590, L593, D594, T595, L596, F598) and also S441 in helix α4 (Fig. 3C–E). We used site directed mutagenesis to determine the functional contribution of these residues in HEK293 cells. CQ-induced Nurr1 transcriptional activity was greatly affected by the following substitutions S441A, I573A, I588A, K590A, L593A, D594A, T595A, L596A, and F598A while mutations of other residues had no effect (Fig. 3F). These results strongly suggest that amino acid residues S441, I573, I588, D594, T595, L596, and F598 contribute to the physical interaction between Nurr1 and CQ and affect CQ-induced transcriptional activation of Nurr1.

### CQ upregulates Nurr1 expression through the CREB signaling pathway

**Figure 4:**
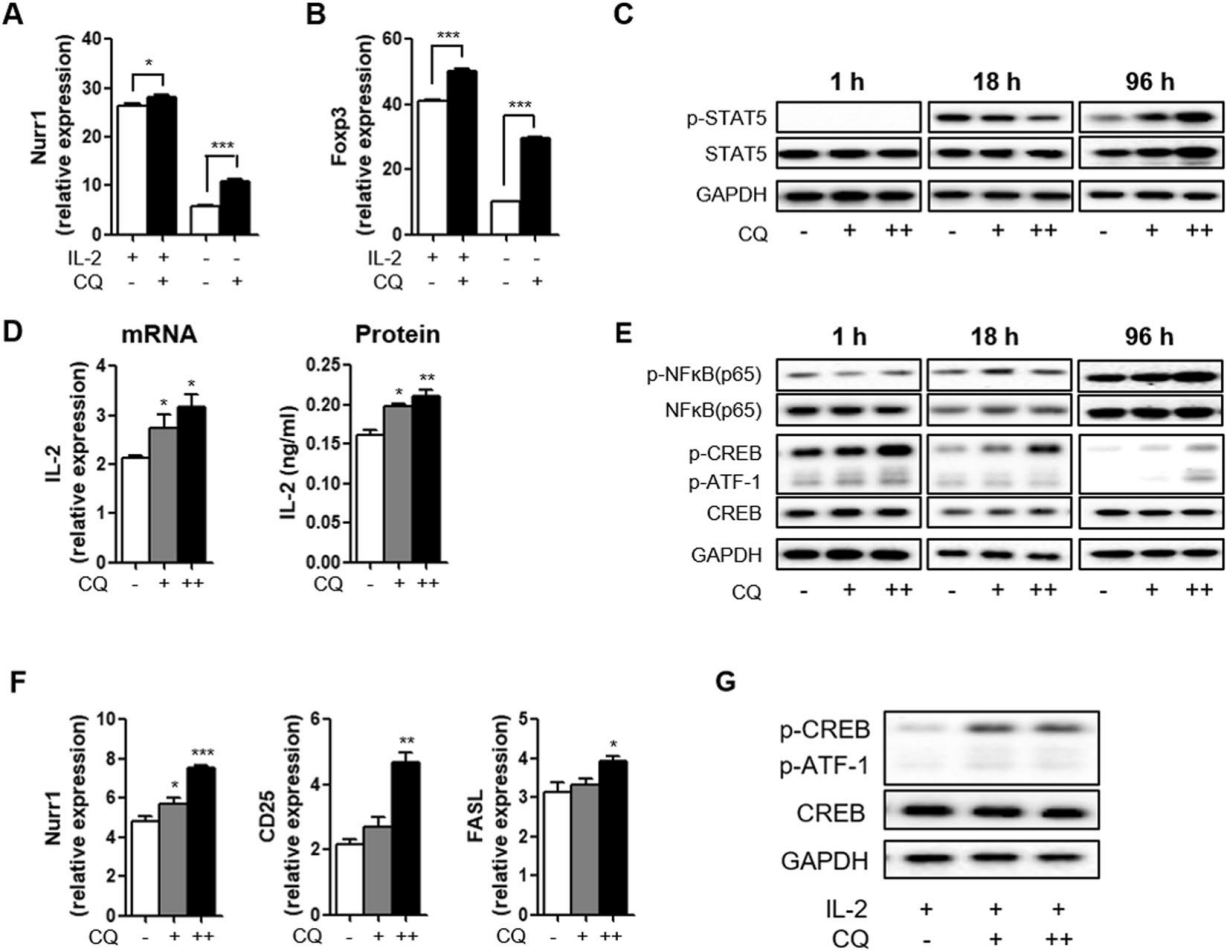
Regulation of Nurr1 expression by CQ. Mouse primary naïve CD4^+^CD25^−^CD62L^high^ T cells were treated with 100 nM (+) or 1 μM (++) CQ and stimulated with plate-bound anti-CD3 and soluble anti-CD28 antibodies for 1–96 h under iT_REG_-polarizing conditions (with or without IL-2). (**A**,**B**) The level of Nurr1 (**A**) and Foxp3 (**B**) mRNA expression were analyzed by quantitative real-time PCR and normalized with GAPDH. (**C**) The expression of p-STAT5, STAT5, and GAPDH proteins were confirmed by western blot after 1, 18, and 96 h treatment with CQ under iT_REG_-polarizing condition without IL-2 treatment. (**D**) The level of IL-2 mRNA and protein expression were analyzed by quantitative real-time PCR and ELISA, respectively. (**E**) The expression of p-NFkB(p65), NFkB(p65), p-CREB, CREB, and GAPDH proteins were confirmed by western blot after 1, 18, and 96 h treatment with CQ under iT_REG_-polarizing condition without IL-2 treatment. (**F**) The level of Nurr1, CD25, and FASL mRNA expression were determined by quantitative real-time PCR and normalized with GAPDH. **(G)** Mouse primary naïve CD4^+^CD25^−^CD62L^high^ T cells were treated with 100 nM (+) or 1 μM (++) CQ and stimulated with plate-bound anti-CD3 and soluble anti-CD28 antibodies for 96 h under iT_REG_-polarizing conditions (with IL-2). The expression of p-CREB, CREB, and GAPDH proteins were confirmed by western blot. These experiments were repeated more than twice in triplicate using independently prepared samples. Each error bar represents means ± s.e.m. *P < 0.05, **P < 0.01, ***P < 0.001.

The above NMR and site-directed mutational studies suggest that CQ can enhance the transcriptional activity of Nurr1, leading to upregulation of Foxp3 gene expression in T_REG_ cells. Interestingly, our results also showed that CQ treatment increases Nurr1 expression (Supplementary Fig. S1A), suggesting that CQ regulates both the function and the expression of Nurr1. To test this possibility, we used *in vitro* iT_REG_-polarizing conditions in the presence and the absence of IL-2, which is critical for this differentiation process via regulating Foxp3 expression^27^. Absence of IL-2 prominently diminished Nurr1 expression (Fig. 4A). However, in the absence of IL-2, treatment with CQ significantly increased both Nurr1 expression (approximately 1.8-fold) and Foxp3 expression (approximately 3.0-fold) (Fig. 4A,B). Based on these results, we tested whether CQ treatment can enhance IL-2-specific signaling pathway. STAT5 is activated through the IL-2/IL-2R-signaling pathway resulting in its phosphorylation (p-STAT5). We did not observe changes in the levels of p-STAT5 at 1 or 18 h post CQ treatment suggesting that CQ does not directly affect the IL-2/IL-2R-signaling pathway. However, at 96 h after CQ treatment we observed increased levels of p-STAT5 (Fig. 4C), suggesting that CQ alters IL-2 expression and then p-STAT5 levels. Indeed, we found that CQ treatment increased the expression of IL-2 at both the mRNA and the protein levels (Fig. 4D).

To investigate how CQ treatment regulates expression of IL-2 and Nurr1 genes, we next tested whether CQ impacts the activities of candidate transcription factors such as NFkB and CREB, which are known to affect IL-2 and Nurr1 expression^28,29^. CQ treatment did not alter the levels of NFkB or of its active phosphorylated form (p-NFkB (p65)) (Fig. 4E). In contrast, CQ treatment increased the phosphorylated form of CREB (p-CREB), in a dose-responsive manner during iT_REG_ differentiation (Fig. 4E). However, as mentioned above, at high concentration, CQ disrupts the activity of p-CREB (Supplementary Fig. S2D). In addition, CQ treatment also significantly up-regulated expression of Nurr1, CD25, and FASL genes, which are known targets of p-CREB (Fig. 4F). These results suggest that CQ can induce IL-2 production but also affect CD25 production, leading to increased IL-2/IL-2R-signaling pathway. We also confirmed that CQ treatment increased the level of p-CREB, in the presence of IL-2 during iT_REG_ differentiation (Fig. 4G). Taken together, these data suggest that CQ treatment activates the CREB pathway, leading to upregulation of Nurr1 and IL-2 expression, promoting iT_REG_ differentiation and associated gene expression.

### CQ suppresses the progression of IBD in DSS-induced mouse model

**Figure 5:**
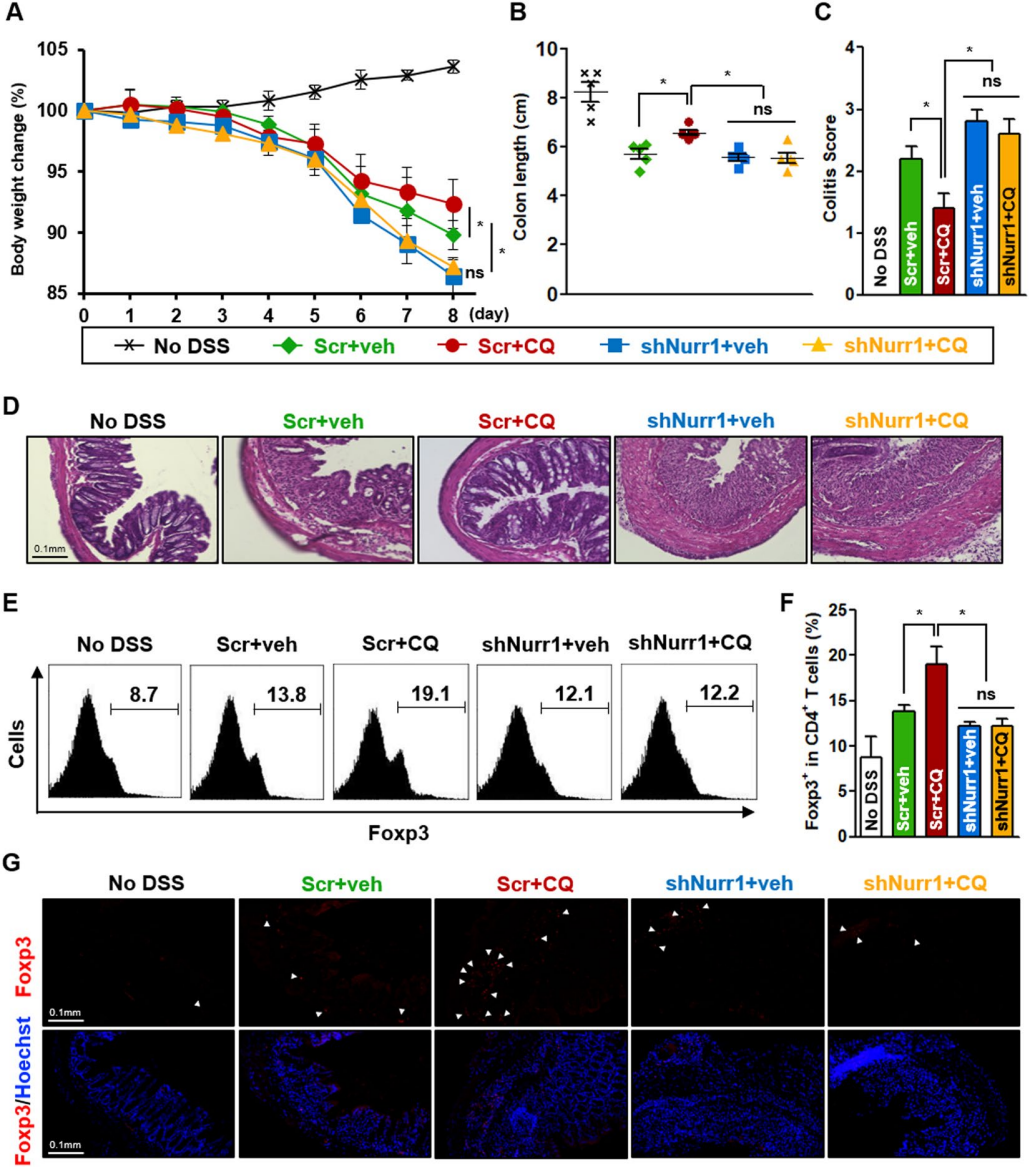
Nurr1-dependent attenuation of DSS-induced colitis by CQ. Male mice were infected with none, scrambled-lentivirus (Scr) or shNurr1-lentivirus (shNurr1) 7 days prior to treatment with water (No DSS) or 3% DSS for 8 days. During the 8 days, each group, Scr + CQ and shNurr1 + CQ, received 50 mg/kg/day of CQ intraperitoneally. Mice were sacrificed at day 8. (**A**) Body weight change after DSS induction of colitis was evaluated and expressed as a percentage of the initial weight. (**B**) Colon length was measured. (**C**) Histological colitis scores were recorded. (**D**) Representative histologic images of H&E-stained colon sections. (**E**) MLN CD4^+^ T cells’ Foxp3 levels were analyzed by flow cytometry. (**F**) Quantification of results in E. (**G**) Representative histologic images of Foxp3/Hoechst-stained colon sections. Data are representative of two experiments with ten mice per group. Each error bar represents means ± s.e.m. *P < 0.05.

We used the DSS-induced colitis mouse model to study how CQ may modulate pathogenic progression and whether it involves Nurr1. Mice were infected with scrambled- or shNurr1-lentivirus and treated with CQ at a concentration known to be effective in this model^30^. We confirmed the ability of shNurr1-lentivirus to knockdown Nurr1 both *in vitro* and *in vivo* (Supplementary Fig. S1C,D). We studied five different groups of mice: No DSS; scrambled-lentivirus infected-DSS fed mice with vehicle injection (Scr + veh) or with CQ injection (Scr + CQ); and shNurr1-lentivirus infected-DSS fed mice with vehicle injection (shNurr1 + veh) or with CQ injection (shNurr1 + CQ) group. All animals treated with DSS showed reduced body weight, shortened colons, and splenomegaly. These changes were significantly attenuated in the CQ-treated group (Fig. 5A,B; Supplementary Fig. S5A,B). Clinical scores were also increased in DSS fed mice, which were significantly reduced in the CQ-treated groups (Fig. 5C). Mice fed with DSS and infected with shNurr1-lentivirus failed to respond to CQ treatment, indicating a relationship between CQ and Nurr1 in the mitigation of IBD in this model (Fig. 5A–C). Histological examination showed that CQ inhibited CD4^+^ T cells infiltration, inflammatory cells and cytokine, and also reduced epithelial cell destruction with goblet cell depletion (Fig. 5D; Supplementary Fig. S5C–E). Notably, this improvement by CQ was not observed in shNurr1-lentivirus infected-DSS fed mice, as evident in comparison of Scr + veh vs. shNurr1 + veh and Scr + CQ vs. shNurr1 + CQ groups. Furthermore, Foxp3 expression and Foxp3-positive cells were significantly up-regulated by CQ treatment in the mesenteric lymph node (MLN) CD4^+^ T cells and colon tissues, respectively (Fig. 5E–G; Supplementary Fig. S5F). Knocking down Nurr1 in shNurr1-lentivirus infected-DSS fed mice prevented the accumulation of Foxp3-positive cells in the MLN and colon tissues further supporting the conclusion that Foxp3 upregulation and functional improvement in this mouse model is Nurr1-dependent.

## Discussion

Autoimmune diseases are associated with an imbalance of pathogenic autoreactive effector T cells and protective regulatory T cells. Many immunotherapies for autoimmune diseases have a common goal of recovering self-tolerance and immune homeostasis through a variety of strategies to regulate immune responses toward dominant T_REG_-mediated regulation^31,32^. Although a variety of drugs with the potential to modify T_REG_- and T_H_17-responses have been developed and approved for the treatment of autoimmune diseases, others are currently being tested in clinical trials. For examples, Norisoboldine, a natural aryl hydrocarbon receptor agonist, promotes T_REG_ differentiation and inhibits collagen-induced arthritis through regulating the balance between T_REG_ and T_H_17 cells^33^. Pioglitazone, a peroxisome proliferator-activated receptor-γ agonist, also attenuated atherosclerotic lesions affecting the T_H_17/T_REG_ balance in an AMPK-dependent mechanism^34^. Low-dose IL-2, a growth factor for T cell proliferation, plays an essential role for T_REG_ differentiation, maintenance and expansion, and a clinical trial is underway to demonstrate its potential as a therapeutic agent for 11 autoimmune diseases^35^. Recently, attempts have been made to find a druggable target that would inhibit the function of T_H_17 cells in order to treat autoimmune diseases. Digoxin, SR1001, or tRORγt-TMD was identified as inverse agonists that interact physically with the putative RORγt-LBD or competitive inhibition of endogenous RORγt^36–38^. As such, a number of drug candidates are currently undergoing clinical studies to treat autoimmune diseases, but they have yet to be approved. Here, we demonstrated that CQ, an FDA-approved drug, can regulate the balance of T_REG_ and T_H_17 cells in a Nurr1-dependent and -independent mechanisms, respectively.

Chloroquine (CQ), developed as an anti-malarial drug almost a century ago, inhibits the parasitic enzyme heme polymerase that converts the toxic heme into non-toxic hemozoin, resulting in the accumulation of toxic heme within the parasite^39^. It was demonstrated that CQ decreases T_H_17-related cytokines from PBMCs of SLE and RA patients^40^ but no mechanism of action was clearly identified. Using high-throughput screening assay systems, we recently identified CQ and two additional FDA-approved drugs (i.e., amodiaquine, and glafenine) that appear to activate the transcriptional activity of Nurr1 via its ligand-binding domain^15^. In the present study, we provide several lines of evidence that support the notion that CQ modulates autoimmune diseases via regulation of Nurr1’s function in T_REG_ cells. First, using NMR spectroscopy we observed that CQ binds to the helix α12 region located at the C-terminal of Nurr1, and confirmed the importance of these interactions using site-directed mutagenesis studies. Nurr1 is considered to be an orphan receptor because its activity appears to be ligand independent and its structure is considered locked in a constitutively active form^41^. However, our results show that the transcriptional activity of Nurr1 can be regulated by CQ through direct binding to Nurr1-LBD, as validated by mutational studies. Second, we demonstrated that CQ increases Nurr1 expression in T cells through CREB, resulting in enhanced Foxp3 expression and T_REG_ cell differentiation. Our results are in line with previous findings showing that ectopic expression of Nurr1 in early phases of T cells differentiation activates Foxp3 expression driving the T_REG_ cell developmental program^14^ and further we identified as-yet unidentified mechanism explaining how CQ increases Nurr1 expression and contributes to T_REG_ cell differentiation. Third, CQ showed inhibitory effects on gene expression and differentiation of pathogenic T_H_17 cells, suggesting that CQ exhibits T cell subset-specific functional effects. However, by confirming that the high concentration of CQ plays a role in inhibiting the function of all T cell subsets including T_REG_, we suggest that there is an effective concentration range for each cell type. It may also be a mechanism related to autophagy, a well-known function of CQ. But it has not been elucidated yet. Finally, using the DSS-induced mouse as an IBD model, we found that CQ can effectively improve symptoms of IBD in a Nurr1-dependent manner, strongly suggesting that the CQ-Nurr1 axis is underlying the CQ’s effect in IBD models.

Various autoimmune disease such as colitis, RA, and SLE are known to be caused by different cell types and mechanisms^42^. Nevertheless, from a T cell perspective, they are commonly induced by T_H_17 cells and treated by T_REG_ cells. Thus, while further studies are warranted to elucidate the detailed mechanisms of CQ in animal models of other forms of autoimmune diseases, our study is the first to offer experimental evidence that CQ’s mechanism of action in the treatment of autoimmune diseases is through its interaction with Nurr1. Our goal here was to identify the mechanism of action of CQ’s in ameliorating autoimmune diseases. Having demonstrated a Nurr1-dependent mechanism of action supports efforts to develop other Nurr1 targeting drugs offering less off target effects. For instance, since prolonged treatment with CQ impairs autophagy and contributes to undesirable side effecs^43^ our results support future efforts to develop better agonists/activators of Nurr1 with less side effects for better treatment of autoimmune diseases such IBD.

In sum, for the first time to our knowledge, we demonstrated that CQ can induce Nurr1 function by two mechanisms: (1) direct binding to Nurr1’s LBD and promoting its transcriptional activity and (2) upregulating Nurr1’s expression, leading to induction of Foxp3 expression and T_REG_ cell differentiation. Since T_REG_ cells are critical to maintain tolerance to self-antigens and for protection against autoimmune diseases^44^, we propose that the induction of Nurr1 function/expression by CQ underlies, at least in part, its effectiveness in the treatment of autoimmune diseases. Importantly, CQ’s functional effect is T cell subset-specific; while it upregulates T_REG_ differentiation and anti-inflammatory cytokine expression it downregulates T_H_17 differentiation and pro-inflammatory cytokine expression, leading to significant protective effects in autoimmune diseases in a Nurr1-dependent manner, as evidenced by our *in vivo* analyses using the DSS-induced colitis mouse model. Taken together, these data show a preclinical “proof of concept” that Nurr1 is a viable effective target for the development of mechanism-based therapies for autoimmune diseases.

## Methods

### Animals

C57BL/6 (8–10 weeks of age, male and female) mice were purchased from The Jackson Laboratory and housed under pathogen-free condition at the Mailman Research Center Animal Care Facility of McLean Hospital. All animal studies were performed in compliance with National Institutes of Health guidelines and were approved by McLean Hospital’s Institutional Animal Care and Use Committee (2015N000002).

### Cell sorting and culture condition

Mouse naïve CD4^+^ T cells were isolated from spleens from C57BL/6 mice on a magnetic-activated cell sorter (MACS) column using CD4, CD25, and CD62L microbeads (Miltenyi Biotec). To isolate CD4^+^ CD25^−^ CD62L^+^ T cells, we performed a three-step purification using CD4-negative beads, CD25-positive beads, and CD62L-positive beads. Primary naïve CD4^+^CD25^−^CD62L^+^ T cells (naïve CD4^+^ T cells) were maintained in complete medium (RPMI1640 containing 10% heat-inactivated FBS, 100 μg/ml penicillin/streptomycin, and 50 mM 2-mercaptoethanol).

### T cell differentiation condition

Naïve CD4^+^ T cells were maintained in complete RPMI1640 medium and stimulated with 1 μg/ml plate-bound anti-CD3 (553057, BD) and 0.5 μg/ml soluble anti-CD28 (553294, BD) under conditions formulated to obtain the following cell types: pathogenic T_H_17 (IL-23 [20 ng/ml], IL-6 [30 ng/ml], and IL-1β [20 ng/ml] with anti-IL-4/IFN-γ (504108/505706, Biolegend) antibody [2 μg/ml, each]), T_H_1 (IL-12 [10 ng/ml] and anti-IL-4 antibody), T_H_2 (IL-4 [40 ng/ml] and anti-IFN-γ antibody), and iT_REG_ (TGF-β1 [5 ng/ml] with or without IL-2 [50 U/ml] with anti-IL-12/IFN-γ antibody [2 μg/ml, each]).

### Measurement of cytokines

After 72–96 h incubation, culture supernatants from stimulated CD4^+^ T cells were analyzed by ELISA for murine IL-2, IL-10, and IL-17A in accordance with the manufacturer’s instructions (eBioscience). After 8 days, DSS mice were euthanized and colon sections were dissected (1.0 cm). Same length of colon sections from each mouse were cultured in complete DMEM media for 24 h and IFN-γ secretion was measured by ELISA from each supernatant.

### RNA isolation and quantitative real-time PCR (qRT-PCR)

Total RNA was isolated from cells with a GeneJET RNA Purification Kit (Thermo Fisher Scientific) according to the manufacturer’s protocol. cDNA synthesis was performed with an iScript^TM^ cDNA Synthesis Kit (Bio-Rad). The following primers were used: RORγt forward: 5′-TGCGACTGGAGGACCTTCTA-3′; reverse: 5′-AGACTGTGTGGTTGTTGGCA-3′, IL17A forward: 5′-CTCCAGAAGGCCCTCAGACTAC-3′; reverse: 5′-AGCTTTCCCTCCGCATTGACACAG-3′, Foxp3 forward: 5′-CCCAGGAAAGACAGCAACCTT-3′; reverse: 5′-TTCTCACAACCAGGCCACTTG-3′, Nurr1 forward: 5′-TTCCAATCCGGCAATGACCA-3′; reverse: 5′-TTACCCTCCACTGGGTTGGA-3′, IL-23R forward: 5′-GCCAAGAGAACCATTCCCGA-3′; reverse: 5′-TCAGTGCTACAATCTTCAGAGGACA-3′, CD25 forward: 5′-AAGTGTGGGAAAACGGGGTG-3′; reverse: 5′-GTGGGTTGTGGGAAGTCTGT-3′, FASL forward: 5′-TGGTGGCTCTGGTTGGAATG-3′; reverse: 5′-GGGTTGGCTATTTGCTTTTCA-3′ and GAPDH forward: 5′-TCAACAGCAACTCCCACTCTTCCA-3′; reverse: 5′-ACCCTGTTGCTGTAGCCGTATTCA-3′. qRT-PCR reactions were performed using the CFX Connect^TM^ Real-Time System (Bio-Rad, Hercules, CA). Calculations of relative expression were performed by using the comparative Ct method, using the naïve CD4^+^ T cell as a reference control (value = 1).

### T_REG_ suppression assays

Conventional CD4^+^ T cells (T_conv_) were labeled with CellTrace^TM^ CFSE (Life Technologies, Invitrogen^TM^) and stimulated with plate-bound anti-CD3 and soluble CD28 antibodies. The same number of naïve CD4^+^ T cells were differentiated into iT_REG_ in the presence of different concentrations of CQ for 96 h (iT_REG_ + CQ, unsorted cells). The resulting iT_REG_ + CQ unsorted cells were added to wells of a 96 well plates seeded with CFSE-labeled T_conv_ cells. The cells were kept in complete medium for 48 h. Natural T_REG_ (nT_REG_, CD4^+^ CD25^+^) cells were isolated by MACS and used as a positive control. CFSE dilution of T_conv_ cells was analyzed by flow cytometry with gating on CFSE-stained CD4^+^ T cells.

### Nuclear magnetic resonance (NMR) data acquisition and assignments

For data acquisition samples contained 0.5–0.6 mM protein in 20 mM sodium phosphate, pH 7.0 with 50 mM NaCl and 0.01% NaN_3_ in 90% H_2_O/10% D_2_O. Backbone resonance assignments were made using transverse relaxation-optimized spectroscopy. TROSY-based HSQC, HNCACB, HN(CO)CACB and HNCA spectra were obtained using fully deuterated and uniformly ^13^C/^15^N-labeled Nurr1-LBD^45^. Post data acquisition, sequence-specific resonance assignments were performed and ~ 94% completeness of all the main backbone ^1^HN, ^15^N, C^α^ and C^β^ atoms were achieved. Molecular interactions between Nurr1-LBD and CQ were determined using two-dimensional TROSY-HSQC spectra of uniformly ^15^N-labeled Nurr1-LBD (0.1–0.2 mM) and 50 mM stock solution of CQ in DMSO changing the molar ratio (Nurr1-LBD: CQ) from 1: 0 to 1: 5. Initial NMR spectrum of the free protein was recorded using 0.2 mM ^15^N-labeled Nurr1-LBD after which CQ was added in accordingly. CQ binding sites were mapped on the crystal structure of Nurr1-LBD (PDB code: 1OVL) by analyzing chemical shift perturbations before and after the addition of CQ. All NMR experiments were performed on a Bruker Avance 700 MHz spectrometer equipped with a 5 mm triple resonance, z-axis-gradient cryogenic probe at 298 K. NMRPipe^46^ was used to process all NMR spectra which were then analyzed using SPARKY 3.113 program (T. D. Goddard and D. G. Kneller, SPARKY 3, University of California).

### Luciferase reporter assays

HEK293 cells were transfected with the following plasmids: pCMV-fNurr1 (mouse full-length Nurr1) or pCMV-fNurr1(mt) which contains mutations in the LBD domain and p4xNL3-Luc. The reporter plasmid, p4xNL3-Luc, contains 4 copies of the NBRE-like NL3 motif inserted upstream of luciferase. Relative luciferase activity was measured using a luciferase assay kit and normalized with β-galactosidase activity.

### Immunoblot analysis

Cells were lysed in RIPA lysis buffer (Sigma) (with protease inhibitors) and protein concentration determined using the BCA assay (Thermo Fisher Scientific). An equal volume of 6X loading buffer was added to each sample, which were then boiled for 10 min and loaded onto a 4–12% Bis-Tris Plus gel. Proteins were electrophoresed and then transferred to polyvinylidene difluoride membrane. The membrane was probed with mouse anti-Foxp3 (ab36607, Abcam), anti-pCREB (9198, CST), anti-CREB (9197, CST), anti-pSTAT5 (9359, CST), anti-STAT5 (94205, CST), anti-pNFkB(p65) (3033, CST), anti-NFkB(p65) (8242, CST) or anti-GAPDH (sc-32233, SCBT) antibody diluted 1:1000 in blocking solution (PBS containing 0.1% BSA) and subsequently incubated with horseradish peroxidase-conjugated secondary antibodies (Amersham). Bound antibodies were visualized using ECL (Amersham).

### Lentivirus production and transduction

Scrambled shRNA (#RHS6848) and specific Nurr1 shRNA cloned into pLKO.1 lentiviral vector were purchased from GE Dharmacon. Knockdown of Nurr1 expression levels was verified by quantitative real-time PCR. Lentiviruses were produced by transfecting HEK293 cells with shRNA plasmid (scrambled or Nurr1) and two helper plasmids (psPAX2 and pMD2.G) using PolyJet^TM^ Reagent (SignaGen Laboratories). The virus-containing medium was harvested 48 h after transfection and then centrifuge at 1,000 rpm for 10 min, and subsequently filtered through a 0.45 μm filter (Millipore). Thereafter, the supernatant was combined with Lenti-X concentrator (Clontech Laboratories) and the mixture was incubated at 4 °C overnight for concentration. After centrifugation (3,000 rpm, 45 min) at 4 °C, the supernatant was carefully removed and then the pellet was suspended in PBS. Finally, the viral titer was determined using the QuickTiter™ Lentivirus Titer Kit (Cell Biolabs) according to the manufacturer’s instruction. Lentivirus transduction into primary T cell was performed with Lentiboost (Sirion Biotech)^47^ at multiplicity of infection (MOI) of 50 or 100, based on toxicity optimization. After 24 h in culture, cells were washed and then stimulated with plate-bound anti-CD3 and soluble anti-CD28 antibodies. After 96 h, cells were harvested and further prepared for RNA and protein isolation.

### Colitis development and histological examination

Mice were infected with ~5 × 10^8^ TU/mouse of scrambled-lentivirus (Scr) or shNurr1-lentivirus (shNurr1) 7 days before treatment. Mice received normal water (No DSS) or 3% dextran sulfate sodium (DSS; MP Biomedicals) water for 8 days and daily injection of CQ (50 mg/kg, ip). Body weight, stool consistency, and blood in the stool were monitored daily. Scoring was as follows: Normal stool with negative hemoccult: 0; Soft stools with positive hemoccult: 1; Very soft stools with traces of blood: 2; Diarrhea with visible rectal bleeding and blood around anus: 3^48^. After 8 days, animals were euthanized, colon length was measured, and colon tissue sections were stained with H&E or Alexa Fluor 488-CD4/Alexa Fluor 568-Foxp3 antibodies.

### Flow cytometry analysis

Cells were isolated from mesenteric lymph node and stained with CD4 antibody (17-0042, eBioscience). To facilitate intracellular staining for Foxp3 (563101, BD), cells were fixed and permeabilized using Foxp3 fixation/permeabilization buffers (BD Biosciences) according to the manufacturer’s instructions.

### Statistical analysis

Microsoft Excel software (Microsoft Corp.) and GraphPad Prism software were used for all statistical analyses and the specific tests used are described in the figure legends. Student’s *t*-test was used when comparing two groups or within a group while multigroup comparisons were performed using two-way ANOVA followed by Bonferroni post-hoc test, or one-way ANOVA followed by Tukey’s test.

## Supplementary information

Supplementary Information
