# Supplementary material for: Chloroquine modulates inflammatory autoimmune responses through Nurr1 in autoimmune diseases
Source: Sci Rep. 2019 Oct 29;9:15559. doi: 10.1038/s41598-019-52085-w (PMC6820774; doi:10.1038/s41598-019-52085-w)

Supplementary Information

Full Title: **Chloroquine modulates inflammatory autoimmune responses through Nurr1 in autoimmune diseases**

Authors: Tae-Yoon Park,[1] Yongwoo Jang,[1] Woori Kim,[1] Joon Shin,[2] Hui Ting Toh,[2] Chun-Hyung Kim,[1] Ho Sup Yoon,[2] Pierre Leblanc,[1,*] Kwang-Soo Kim[1,3,*]

Affiliations: [1]Molecular Neurobiology Laboratory, Department of Psychiatry and McLean Hospital, Harvard Medical School, 115 Mill Street, Belmont, Massachusetts 02478, USA

[2]School of Biological Sciences, Nanyang Technological University, 50 Nanyang Avenue, Singapore 639798

[3]Program in Neuroscience and Harvard Stem Cell Institute, McLean Hospital, Harvard Medical School, Belmont, MA 02478

Supplementary Figure S1.

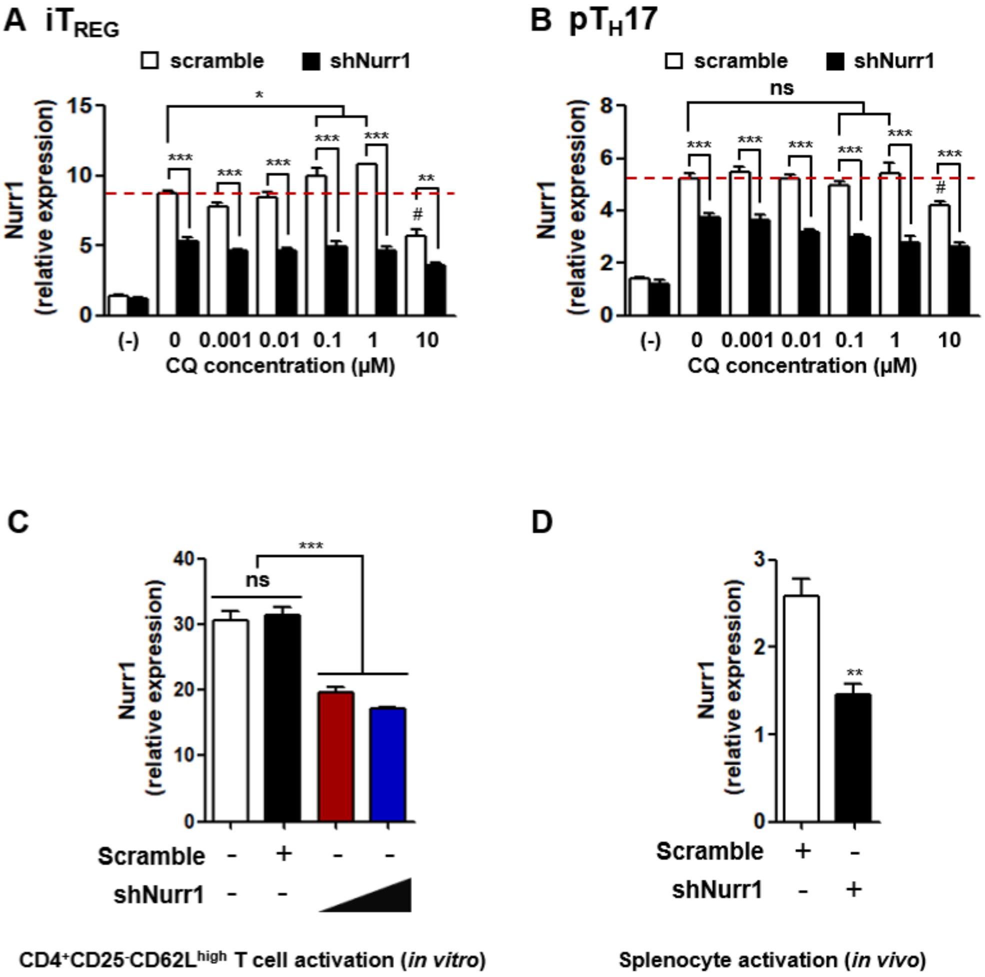

Supplementary Figure S1 *In vitro* and *in vivo* Nurr1-knockdown effect by shNurr1-lentiviral vector transfection or shNurr1-lentivirus. $CD4^{+}CD25^{-}CD62L^{high}$ T cells were transfected with lenti-scramble- or lenti-shNurr1-plasmid. Cells were treated with CQ (0.001 ~ 10 μM) and stimulated with plate-bound anti-CD3 and soluble anti-CD28 antibodies for 96 h under iT_{REG} **(A)** or $pT_{H}17$ **(B)**-polarizing conditions. The level of Nurr1 mRNA expression was determined by quantitative real-time PCR and normalized with GAPDH. **(C)** Mouse primary naïve $CD4^{+}CD25^{-}CD62L^{high}$ T cells were infected with scrambled-lentivirus (100 moi) or shNurr1-lentivirus (50, 100 moi) for 24 h and then stimulated with plate-bound anti-CD3 and soluble anti-CD28 antibodies for 96 h. The level of Nurr1 mRNA expression was analyzed by quantitative real-time PCR and normalized with GAPDH. **(D)** C57BL/6 male mice were infected with the same dose of scrambled-lentivirus or shNurr1-lentivirus used in the DSS-induced colitis experiment for 7 days. Splenocytes from each mouse were stimulated with plate-bound anti-CD3 and soluble anti-CD28 antibodies for 96 h. The level of Nurr1 mRNA expression was determined by quantitative real-time PCR. These experiments were repeated three times in triplicate using independently prepared samples. Each error bar represents means ± s.e.m. ***, $P < 0.001$, #, $P < 0.05$, # compared with the CQ-untreated and lenti-scramble transfected sample.

Supplementary Figure S2.

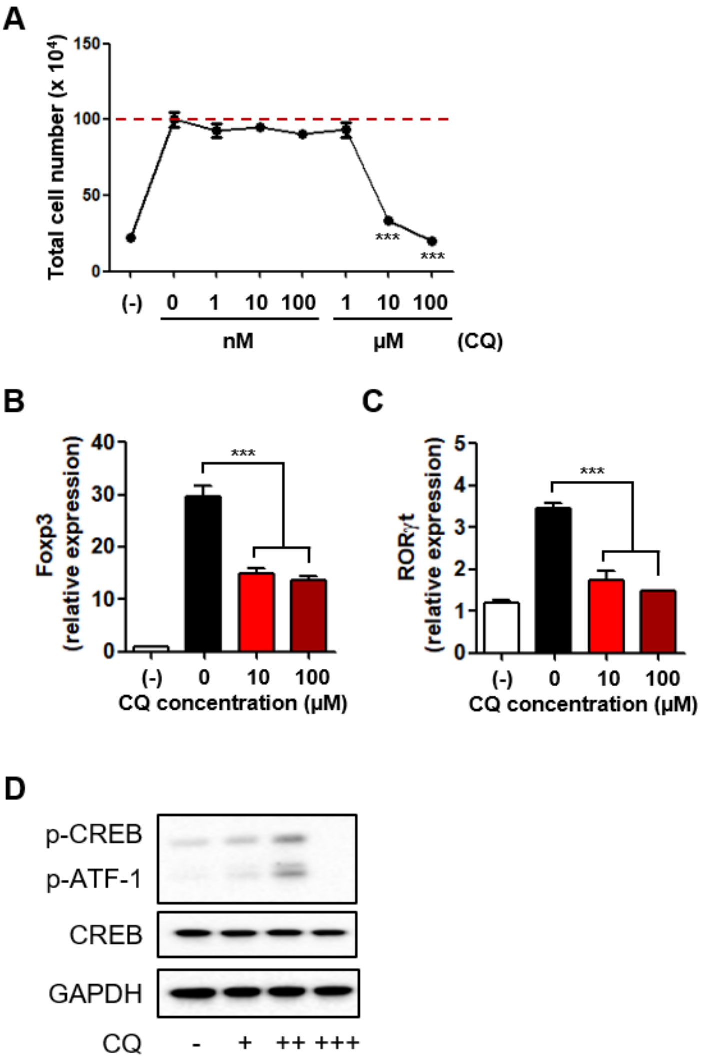

Supplementary Figure S2. Effect of treatment with high concentration of CQ. (**A**) Mouse primary naïve $CD4^{+}CD25^{-}CD62L^{high}$ T cells were treated with 1 nM ~ 100 μM CQ and activated with plate-bound anti-CD3 and soluble anti-CD28 antibodies for 72 h. Total cell numbers were counted. (**B, C**) Naïve T cells were treated with 10 or 100 μM CQ and differentiated under iT_{REG} or $pT_{H}17$-polarizing conditions. The levels of Foxp3 (**B**) and RORγt (**C**) mRNA expression were determined by quantitative real-time PCR and normalized with GAPDH. (**D**) Naïve T cells were treated with 100 nM (+), 1 μM (++) or 10 μM (+++) CQ and stimulated with plate-bound anti-CD3 and soluble anti-CD28 antibodies for 96 h under iT_{REG}-polarizing conditions (without IL-2). The expression of p-CREB, CREB, and GAPDH proteins were confirmed by western blot. Each error bar represents means ± s.e.m. ***, $P < 0.001$.

Supplementary Figure S3.

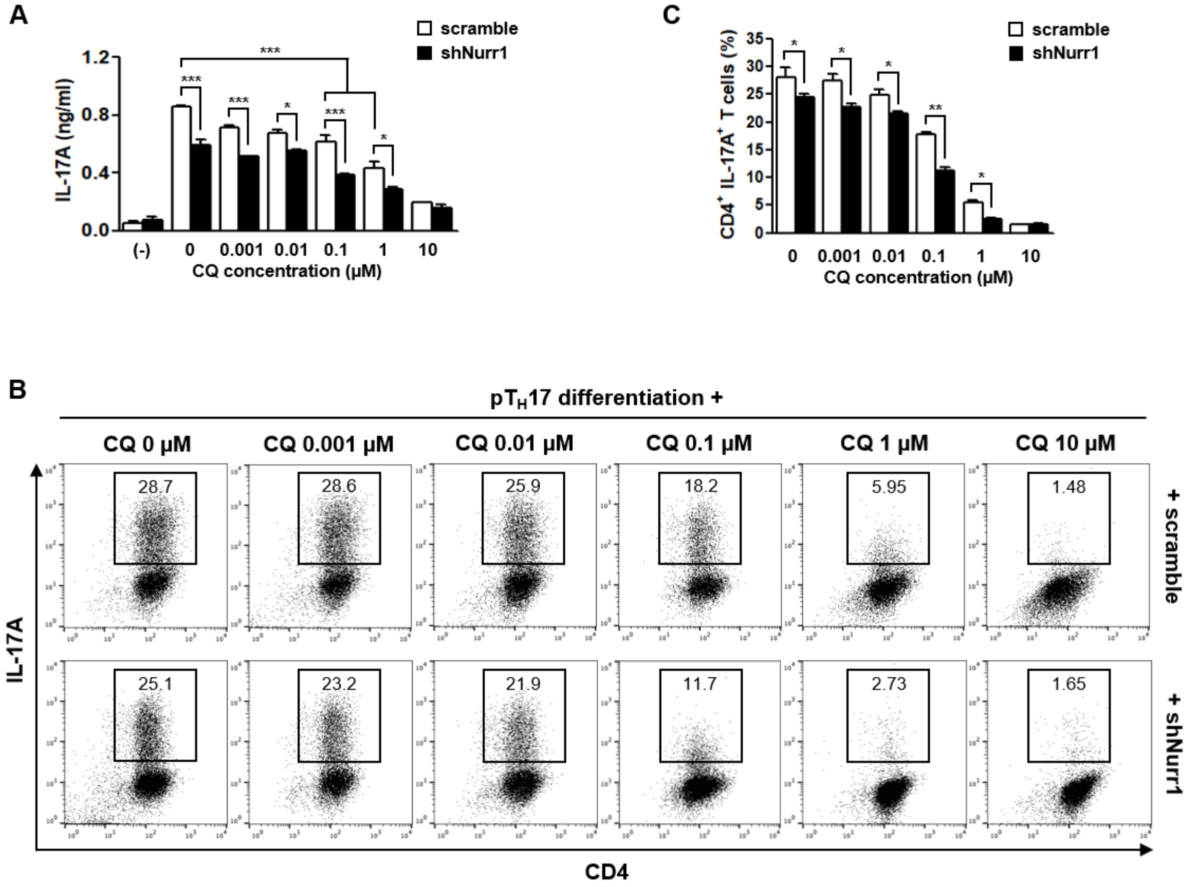

Supplementary Figure S3 Nurr1-independent regulation of pT_H17 differentiation by CQ. Mouse primary naïve $CD4^{+}CD25^{-}CD62L^{high}$ T cells were transfected with lenti-scramble- or lenti-shNurr1-plasmid. Cells were treated with CQ (0.001 ~ 10 μM) and stimulated with plate-bound anti-CD3 and soluble anti-CD28 antibodies for 72 h under pT_H17-polarizing conditions. (**A**) The level of IL-17A in the culture media was analyzed by ELISA. (**B**) Differentiated pT_H17 cells were re-stimulated with PMA/ionomycin and stained with anti-CD4 and anti-IL-17A, analyzed with flow cytometry. (**C**) Quantification of results in B. These experiments were repeated two or three times in triplicate using independently prepared samples. Each error bar represents means ± s.e.m. *, $P < 0.05$, **, $P < 0.01$, ***, $P < 0.001$.

Supplementary Figure S4.

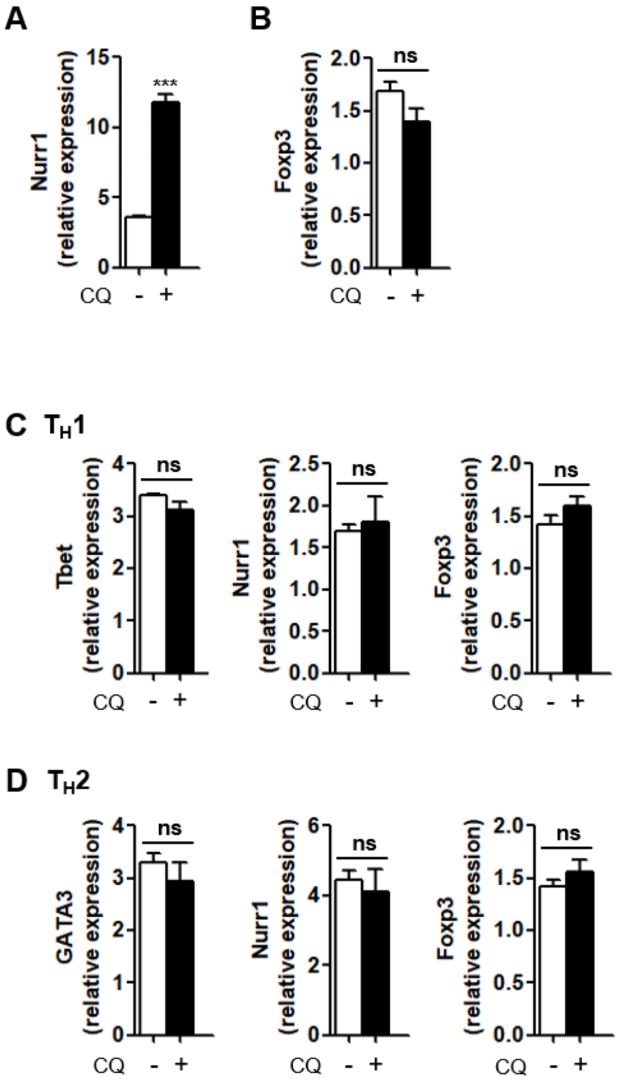

Supplementary Figure S4. Effect of CQ on Nurr1 and Foxp3 expression in activated T cells and under T_H1 and T_H2 cell differentiation conditions. **(A, B)** Mouse primary naïve $CD4^+CD25^-$ $CD62L^{high}$ T cells were treated with 100 nM CQ and stimulated with plate-bound anti-CD3 and soluble anti-CD28 antibodies for 72 h. The levels of Nurr1 **(A)** and Foxp3 **(B)** mRNA expression were determined by quantitative real-time PCR and normalized with GAPDH. **(C, D)** Mouse primary naïve $CD4^+CD25^-CD62L^{high}$ T cells were treated with 100 nM of CQ and stimulated with plate-bound anti-CD3 and soluble anti-CD28 antibodies for 72 h under T_H1 and T_H2 -polarizing conditions. The levels of Tbet, Nurr1, Foxp3 **(C)** and GATA-3, Nurr1, Foxp3 **(D)** mRNA expression were determined by quantitative real-time PCR and normalized with GAPDH. This experiment was repeated three times in triplicate using independently prepared samples. Each error bar represents means ± s.e.m. ***, $P < 0.001$.

Supplementary Figure S5.

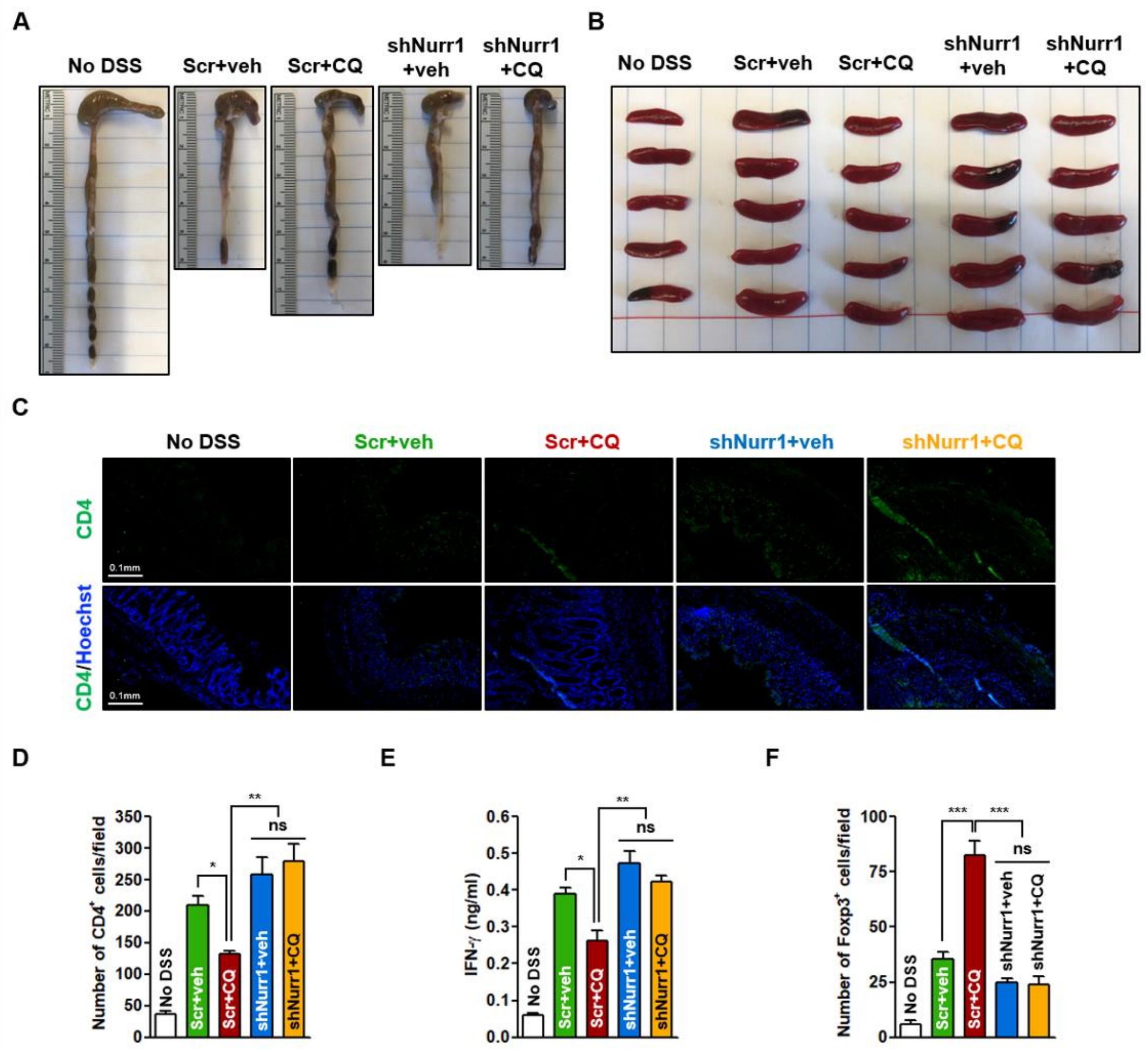

Supplementary Figure S5. Nurr1-dependent attenuation of DSS-induced colitis by CQ. Colons shapes and lengths (**A**) and spleens sizes (**B**) were examined. **(C)** Representative histologic images of CD4/Hoechst-stained colon sections. **(D)** Quantification of results in S4C. **(E)** Colon tissues were dissected and incubated in DMEM media, and supernatants were used for measuring IFN-γ secretion by ELISA. **(F)** Quantification of results in Figure 5G. Data are representative of two experiments with ten mice per group. Each error bar represents means ± s.e.m. *, $P < 0.05$, **, $P < 0.01$, ***, $P < 0.001$.

Uncropped blots in Figure 1B.

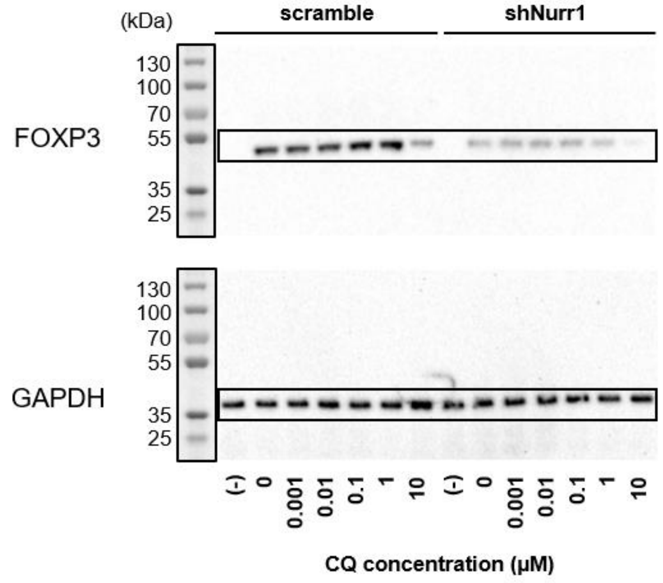

Uncropped blots in Figure 4C.

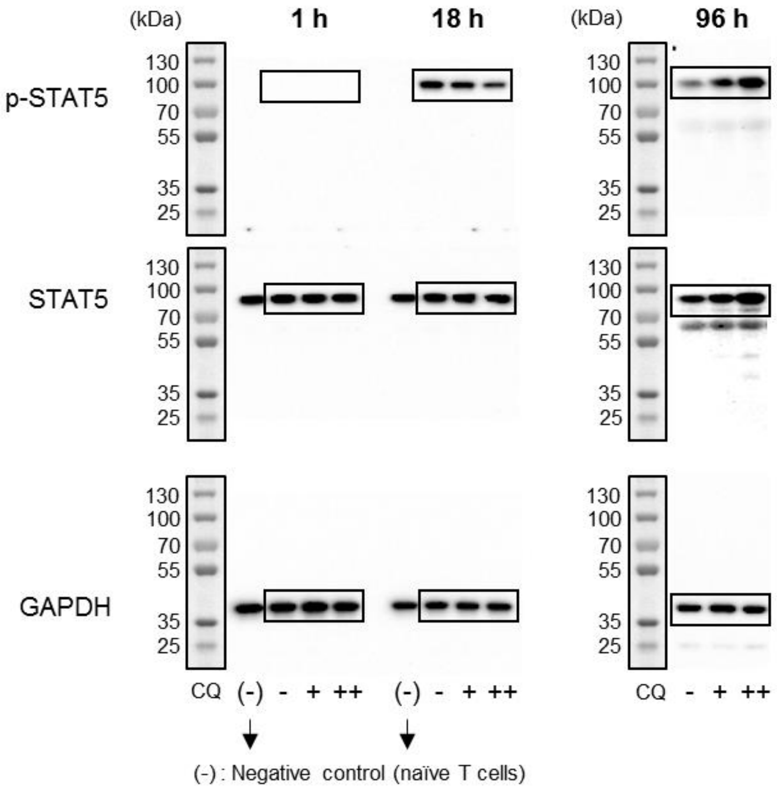

Uncropped blots in Figure 4E.

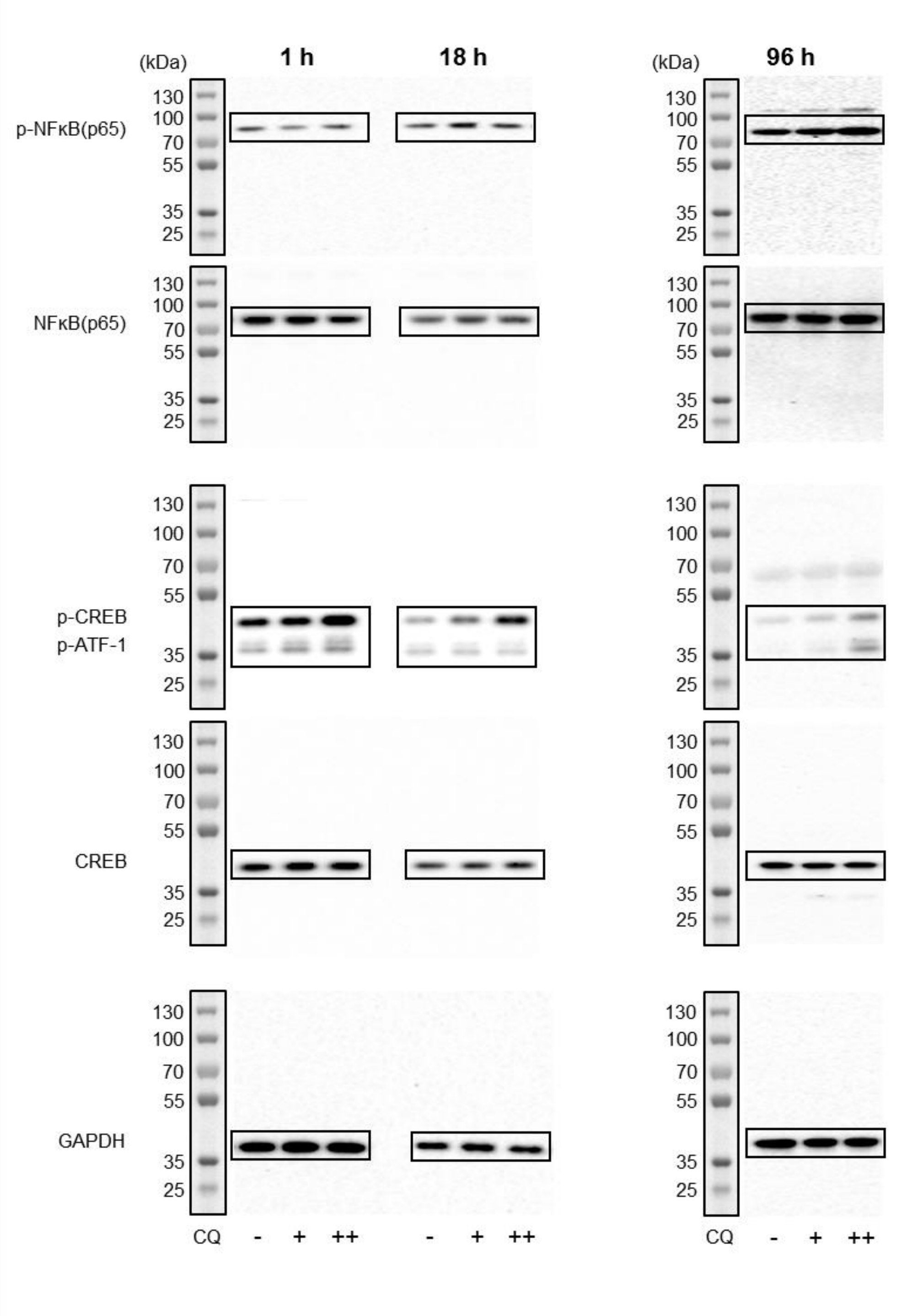

Uncropped blots in Figure 4G.

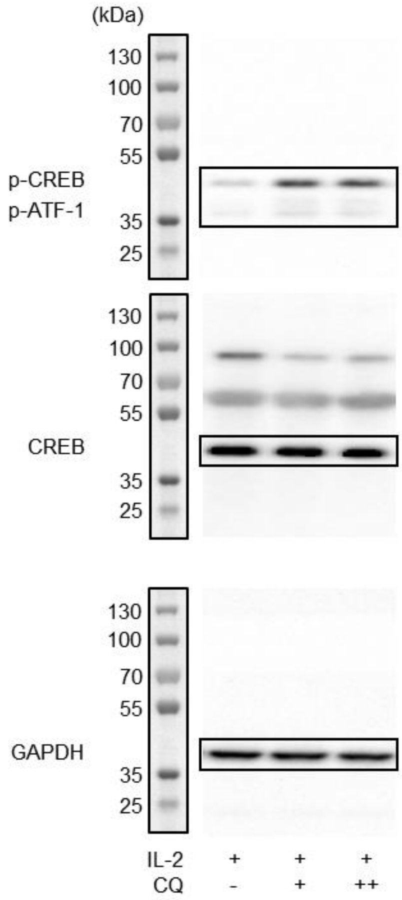

Uncropped blots in Supplementary Figure S2D.

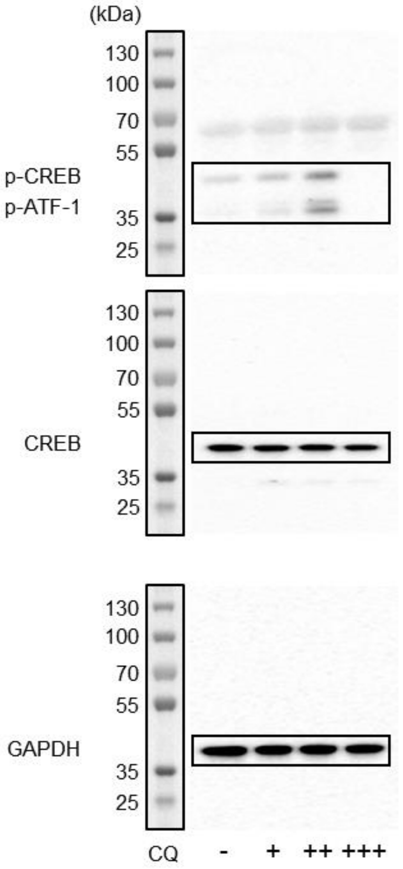

Supplement: Supplementary file 1 — Supplementary Information [file 41598_2019_52085_MOESM1_ESM.pdf]
